# Association of Health Insurance Coverage Disruptions With Mortality Risk Among US Working-Age Adults

**DOI:** 10.1001/jamahealthforum.2022.4258

**Published:** 2022-11-23

**Authors:** K. Robin Yabroff, Xuesong Han, Jingxuan Zhao, James Kirby, Leticia Nogueira, Zhiyuan Zheng

**Affiliations:** 1 Surveillance and Health Equity Science, American Cancer Society, Kennesaw, Georgia; 2 Center for Financing, Access and Cost Trends, The Agency for Healthcare Research and Quality, Rockville, Maryland

## Abstract

This cohort study assesses associations of a prior coverage disruption with mortality risk among large, nationally representative cohorts of working-age adults with public or private health insurance coverage.

## Introduction

Having health insurance coverage is strongly associated with better access to care and health outcomes in the US.^1,2^ Accumulating evidence suggests that health insurance coverage disruptions—periods without insurance—are associated with lacking a usual source of care^3^ and delaying or forgoing care due to cost.^4^ Most research has been conducted among Medicaid enrollees; little is known about health effects of coverage disruptions among privately insured adults, the majority of the working-age population. In this study, we evaluated associations of a prior coverage disruption with mortality risk among large, nationally representative cohorts of working-age adults with either public or private health insurance coverage.

## Methods

In this cohort study, adults aged 18 to 64 years with either private or public health insurance were identified from the 2000 to 2018 National Health Interview Survey (NHIS). The NHIS Linked Mortality files^5^ contain data from the National Death Index; year and quarter of death were available for decedents, and all others were censored at the end of follow-up, December 31, 2019. This article followed the Strengthening the Reporting of Observational Studies in Epidemiology (STROBE) reporting guideline for cohort studies. All NHIS and NHIS Linked Mortality files were deidentified and publicly available and not considered human participant research by an institutional review board based on National Institutes of Health guidance.

Coverage disruption was measured from the question: “In the past 12 months, was there any time when you did not have any health insurance or coverage?” Sample characteristics were compared by coverage type and disruption status using Wald χ^2^ tests. Survival curves were evaluated visually to ensure proportionality, and weighted Cox proportional hazards models were used to estimate crude and adjusted hazard ratios (HR) for associations of coverage disruptions with mortality using age as the time scale.^5^ Sensitivity analyses used propensity score matching to select controls balanced on covariates (eMethods in the Supplement). Race and ethnicity are reported to describe the diversity of the sample.

Statistical analyses were performed with SAS statistical software, version 9.4 (SAS Institute Inc) and conducted from March to September 2022. Comparisons were 2-sided with significance as *P* < .05.

## Results

The sample consisted of adults with either private (n = 246 622; 50.56% female; median [IQR] age, 41.29 [29.96-51.52] years) or public (n = 47 473; 61.00% female; median [IQR] age, 35.68 [24.70-49.04] years) insurance at the NHIS interview; 4.9% and 12.8% reported a prior disruption, respectively (Table 1). During the study period, there were 9686 (3.93%) and 3590 (7.56%) deaths among adults with private and public coverage, respectively. Among adults with private coverage, disruptions were associated with higher mortality risk in adjusted analyses of the full (HR, 1.63; 95% CI, 1.45-1.83) and in the propensity score matched (HR, 1.41; 95% CI, 1.26-1.59) samples, compared with continuous coverage (Table 2). Among adults with public coverage, disruptions were associated with higher mortality risk in adjusted analyses of the full (HR, 1.15; 95% CI, 1.01-1.30) and matched (HR, 1.22; 95% CI, 1.07-1.39) samples, compared with continuous coverage.

**Table 1.** Sample Characteristics by Current Health Insurance Coverage and Prior Coverage Disruption Within 12 Months Among Adults Aged 18 to 64 Years^a^

Characteristic	Publicly insured at NHIS interview			Privately insured at NHIS interview		
	With coverage disruption (n = 5983) [average follow-up: 8.84 y]		Without coverage disruption (n = 41 490) [average follow-up: 8.32 y], %	With coverage disruption (n = 13 108) [average follow-up: 9.56 y]		Without coverage disruption (n = 233 514) [average follow-up: 9.73 y], %
	Weighted %	*P* value		Weighted %	*P* value	
Overall weighted value of 4 groups, %	1.8	NA	12.0	4.2	NA	82.0
Weighted value by insurance type, %	12.8	NA	87.2	4.9	NA	95.1
Age group, y						
18-29	43.4	<.001^b^	34.3	39.5	<.001^b^	22.2
30-39	24.2		20.5	25.1		21.4
40-49	16.6		18.9	19.1		24.5
50-64	15.8		26.4	16.3		31.9
No. of health conditions^c^						
0	52.5	<.001^b^	51.0	61.8	<.001^b^	60.2
1	28.3		26.2	26.7		26.6
≥2	19.1		22.8	11.5		13.2
Sex						
Female	65.9	<.001^b^	60.3	50.1	.410	50.6
Male	34.1		39.7	49.9		49.4
Race/ethnicity						
Hispanic	21.1	<.001^b^	21.3	13.9	<.001^b^	9.9
Non-Hispanic Black	19.1		22.2	12.1		9.7
Non-Hispanic White	54.8		50.1	68.3		74.2
Other^d^	4.9		6.5	5.6		6.2
Current marital status						
Married	49.8	.048^b^	47.9	57.6	<.001^b^	69.0
Not married^e^	50.2		52.1	42.4		31.0
Education						
Less than high school	25.7	.367	26.9	8.8	<.001^b^	6.4
High school graduate	33.3		32.4	24.8		23.1
Some college or more	40.9		40.7	66.4		70.5
Region						
Northeast	19.0	<.001^b^	20.1	14.2	<.001^b^	19.1
Midwest	24.9		20.0	24.6		25.5
South	30.4		33.9	37.6		34.3
West	25.8		26.0	23.6		21.1
Era (survey y)						
2001-2005	19.2	.002^b^	19.2	26.3	<.001^b^	28.2
2006-2009	22.6		20.7	23.7		23.3
2010-2013	27.1		25.9	22.9		23.3
2014-2018	31.0		34.3	27.1		25.1

Abbreviation: NA, not applicable.

^a^ Data were derived from the 2001 to 2018 National Health Interview Survey (NHIS). The sample was restricted to adults with known vital status as of December 31, 2019, and eligible for survival analysis. Average follow-up for the full sample of adults with either public or private insurance coverage at the NHIS interview through December 31, 2019, was 9.46 years. Average follow-up for adults publicly insured and privately insured at the NHIS interview was 8.37 years and 9.72 years, respectively.

^b^ Value is statistically significant (*P* < .05).

^c^ Conditions included arthritis, asthma, cancer, diabetes, emphysema, heart disease (angina, coronary heart disease, heart attack, other heart condition/disease), high cholesterol, hypertension, and stroke.

^d^ Other races and ethnicities include Asian, Hispanic Black, Native American and Alaska Native, multiple races, and unknown.

^e^ Not married includes widowed, divorced, separated, or never married.

**Table 2.** Association of Health Insurance Coverage Disruptions in Past 12 Months With Mortality Risk Among Adults Aged 18 to 64 Years^a^

Characteristic	Crude model		Adjusted model		Propensity score matched model^b^		
	Weighted HR (95% CI)	*P* value	Weighted HR (95% CI)	*P* value	Unweighted HR (95% CI)		*P* value
**Private health insurance coverage at interview**							
Prior health insurance coverage disruption							
Continuous coverage	1 [Reference]	NA	1 [Reference]	NA	1 [Reference]		NA
Coverage disruption	1.78 (1.59-1.99)	<.001	1.63 (1.45-1.83)	<.001	1.41 (1.26-1.59)		<.001
No. of health conditions^c^							
0	1 [Reference]	NA	1 [Reference]	NA	NA	NA	
1	1.04 (0.98-1.10)	.19	1.06 (1.00-1.12)	.06	NA	NA	
≥2	1.66 (1.57-1.75)	<.001	1.65 (1.56-1.75)	<.001	NA	NA	
Sex							
Female	1 [Reference]	NA	1 [Reference]	NA	NA	NA	
Male	1.58 (1.50-1.66)	<.001	1.67 (1.59-1.75)	<.001	NA	NA	
Current marital status							
Married	1 [Reference]	NA	1 [Reference]	NA	NA	NA	
Not married^d^	1.52 (1.45-1.60)	<.001	1.63 (1.55-1.71)	<.001	NA	NA	
Education							
Some college or more	1 [Reference]	NA	1 [Reference]	NA	NA	NA	
High school graduate	1.48 (1.40-1.56)	<.001	1.43 (1.35-1.50)	<.001	NA	NA	
Less than high school	1.79 (1.67-1.92)	<.001	1.62 (1.51-1.74)	<.001	NA	NA	
Region							
Northeast	1 [Reference]	NA	1 [Reference]	NA	NA	NA	
Midwest	1.18 (1.09-1.28)	<.001	1.16 (1.07-1.25)	<.001	NA	NA	
South	1.25 (1.16-1.35)	<.001	1.22 (1.14-1.32)	<.001	NA	NA	
West	0.96 (0.88-1.05)	.96	1.00 (0.92-1.09)	.96	NA	NA	
Era (survey y)							
2001-2005	1 [Reference]	NA	1 [Reference]	NA	NA	NA	
2006-2009	0.86 (0.80-0.92)	<.001	0.84 (0.79-0.90)	<.001	NA	NA	
2010-2013	0.73 (0.68-0.79)	<.001	0.72 (0.67-0.78)	<.001	NA	NA	
2014-2018	0.45 (0.40-0.50)	<.001	0.43 (0.38-0.48)	<.001	NA	NA	
**Public health insurance coverage at interview**							
Prior health insurance coverage disruption							
Continuous coverage	1 [Reference]	NA	1 [Reference]	NA	1 [Reference]		NA
Coverage disruption	1.11 (0.98-1.26)	.10	1.15 (1.01-1.30)	.03	1.22 (1.07-1.39)		.002
No. of health conditions^c^							
0	1 [Reference]	NA	1 [Reference]	NA	NA	NA	
1	0.94 (0.84-1.05)	.25	0.95 (0.85-1.06)	.33	NA	NA	
≥2	1.39 (1.28-1.52)	<.001	1.36 (1.25-1.48)	<.001	NA	NA	
Sex							
Female	1 [Reference]	NA	1 [Reference]	NA	NA	NA	
Male	1.32 (1.24-1.41)	<.001	1.45 (1.36-1.54)	<.001	NA	NA	
Current marital status							
Married	1 [Reference]	NA	1 [Reference]	NA	NA	NA	
Not married^d^	1.55 (1.45-1.66)	<.001	1.59 (1.48-1.70)	<.001	NA	NA	
Education							
Some college or more	1 [Reference]	NA	1 [Reference]	NA	NA	NA	
High school graduate	1.41 (1.30-1.52)	<.001	1.35 (1.24-1.46)	<.001	NA	NA	
Less than high school	1.49 (1.37-1.62)	<.001	1.36 (1.25-1.47)	<.001	NA	NA	
Region							
Northeast	1 [Reference]	NA	1 [Reference]	NA	NA	NA	
Midwest	1.29 (1.17-1.43)	<.001	1.27 (1.15-1.40)	<.001	NA	NA	
South	1.20 (1.09-1.32)	<.001	1.21 (1.10-1.33)	<.001	NA	NA	
West	0.93 (0.83-1.03)	.17	1.00 (0.90-1.12)	.95	NA	NA	
Era (survey y)							
2001-2005	1 [Reference]	NA	1 [Reference]	NA	NA	NA	
2006-2009	0.95 (0.87-1.03)	.20	0.92 (0.84-1.00)	.04	NA	NA	
2010-2013	0.73 (0.67-0.80)	<.001	0.71 (0.65-0.78)	<.001	NA	NA	
2014-2018	0.51 (0.46-0.57)	<.001	0.49 (0.45-0.55)	<.001	NA	NA	

Abbreviations: HR, hazard ratio; NA, not applicable.

^a^ Data were derived from the 2001 to 2018 National Health Interview Survey. The sample was restricted to adults with known vital status as of December 31, 2019, and eligible for survival analysis. Prior coverage disruption was defined as no health insurance coverage sometime in the 12 months before the interview.

^b^ Adults with coverage disruptions were propensity score matched to adults without coverage disruptions in a 1:2 ratio on age group at the time of survey, survey weight, number of health conditions, sex, marital status, educational attainment, and survey year era. The multivariable proportional hazards analyses from the propensity score matched sample did not use sample weights.

^c^ Conditions included arthritis, asthma, cancer, diabetes, emphysema, heart disease (angina, coronary heart disease, heart attack, other heart condition/disease), high cholesterol, hypertension, and stroke.

^d^ Not married includes widowed, divorced, separated, or never married.

## Discussion

In this large, nationally representative cohort study, prior health insurance coverage disruptions were associated with higher mortality risk among US working-age adults with either private or public health insurance coverage. These findings suggest that assessment of prior insurance coverage disruptions may be an important addition to social risk screening in clinical practice.

This study’s findings are timely with some states debating Medicaid expansions under the Affordable Care Act; earlier research demonstrated that Medicaid expansions were associated with reductions in coverage disruptions,^6^ better care access, and outcomes. The recently passed Inflation Reduction Act extends Marketplace subsidies through 2025, which may reduce disruptions in private coverage during these years.

This study has limitations, including self-reported insurance coverage, comorbidity, and socioeconomic status measured once at the time of the survey; earlier or later coverage disruptions and reasons for coverage disruptions (eg, development of serious health conditions, job loss, or other economic shocks) were unavailable. Additional research evaluating temporal associations between coverage disruptions, reasons for coverage gains or losses, development of health conditions, and changes in socioeconomic status is warranted.

In summary, among working-age adults with either private or public health insurance, a prior coverage disruption was associated with increased mortality risk, underscoring the need to assess health insurance coverage instability in clinical practice.

## Notes

Supplement. **eMethods. **Association of health insurance coverage disruptions with mortality risk among US working-age adults supplemental methods
