# Supplementary material for: Association of Health Insurance Coverage Disruptions With Mortality Risk Among US Working-Age Adults
Source: JAMA Health Forum. 2022 Nov 23;3(11):e224258. doi: 10.1001/jamahealthforum.2022.4258 (PMC9685485; doi:10.1001/jamahealthforum.2022.4258)
Supplement: Supplement. — eMethods. Association of health insurance coverage disruptions with mortality risk among US working-age adults supplemental methods [file jamahealthforum-e224258-s001.pdf]

Supplemental Online Content

Yabroff KR, Han X, Zhao J, Kirby J, Nogueira L, Zheng Z. Association of health insurance coverage disruptions with mortality risk among US working-age adults. *JAMA Health Forum.* 2022;3(11):e224258. doi:10.1001/jamahealthforum.2022.4258

eMethods. Association of health insurance coverage disruptions with mortality risk among US working-age adults supplemental methods

This supplemental material has been provided by the authors to give readers additional information about their work.

© 2022 Yabroff KR et al. *JAMA Health Forum.*

eMethods. Association of health insurance coverage disruptions with mortality risk among US working-age adults supplemental methods

This study examined association of health insurance coverage disruptions with mortality risk in working-age adults using data from the 2000-2018 National Health Interview Survey (NHIS) and NHIS Linked Mortality files with follow up of vital status through December 31, 2019. The main analytic approach used weighted Cox proportional hazards models to incorporate complex survey design and estimate crude and adjusted hazard ratios (HR) for associations of coverage disruptions and mortality, separately for adults with either private or public health insurance at the NHIS interview. Although multivariable modeling is a standard approach to minimizing confounding bias due to measured and unmeasured patient characteristics associated with both exposure (coverage disruptions) and study outcome (mortality) in observational studies, multivariable modeling can be limited if covariate distributions across patient groups are unbalanced. In sensitivity analysis, we used a propensity score matching approach to balance the observable patient characteristics across groups with and without health insurance coverage disruptions, separately for adults with either public or private health insurance coverage at the NHIS interview.

The propensity scores of reporting a health insurance coverage disruption in the 12 months prior to the survey and the corresponding matched sample were generated by the PSMATCH procedure (SAS 9.4). Sample characteristics used for matching included age group at the time of survey, survey weight, number of health conditions, sex, marital status, educational attainment, and survey year era. These covariates were used in multivariable Cox proportional hazards models. The "greedy match" method was used to conduct the 1:2 matching, which finds the best

© 2022 Yabroff KR et al. *JAMA Health Forum.*

initial 1:1 match with the smallest within-pair propensity score difference among all available pairs between the treated unit (with prior insurance disruption) and the control (without prior insurance disruption) unit. The second round tried to find another nearest control unit from the remaining control group for each treated unit that was matched in the first round. Both treated units matched to only 1 control unit and those matched to 2 control units were included in the final matched analysis if the within-pair propensity score difference was less than 0.001. Of note, analyses using the propensity matched sample were sensitive to matching criteria and variable selection in generating the propensity scores because of variation in the NHIS sample weights.

As shown in the accompanying Table, covariate distributions for adults with and without health insurance coverage disruptions for groups with private or public coverage at the NHIS interview were well-balanced following propensity score matching. However, an important limitation of propensity score matching is that it may not be possible to identify controls with the same covariates used for matching for all individuals in the group with the exposure of interest. This problem is more common at the extremes of any covariate distribution and a downside of propensity score matching is that it can introduce bias by excluding some individuals without matching controls. Of note, there were 65 fewer individuals with public health insurance coverage at interview and a disruption in the past 12 months in the propensity score matched sample than in the full sample.

© 2022 Yabroff KR et al. *JAMA Health Forum.*

Propensity score matched sample characteristics* by health insurance coverage at interview and coverage disruption within 12 months prior to interview, ages 18-64 years, 2001-2018 National Health Interview Survey							
	**Publicly Insured at NHIS Interview**				**Privately insured at NHIS Interview**		
	**With coverage disruption (N = 5,918)**		**Without coverage disruption (N = 11,657)**		**With coverage disruption (N = 13,034)**		**Without coverage disruption (N = 25,960)**
	**Unweighted %**	**P-value**	**Unweighted %**		**Unweighted %**	**P-value**	**Unweighted %**
**Age group**		0.995				1.0	
18-29	38.8		38.7		36.5		36.5
30-39	26.0		25.9		26.3		26.3
40-49	17.5		17.5		19.3		19.3
50-64	17.8		17.9		17.9		17.9
**Number of health conditions ‖**		0.807				0.213	
0	51.6		51.6		61.9		61.7
1	27.9		27.9		26.4		26.0
2+	20.5		20.5		11.7		12.3
**Sex**		0.893				0.478	
Female	70.6		70.7		53.4		53.8
Male	29.4		29.3		46.6		46.2
**Current marital status**		0.417				0.276	
Married	40.7		40.1		47.5		46.9
Not married†	59.3		59.9		52.5		53.1
**Education**		0.394				0.505	
Less than high school	26.7		25.8		8.8		8.6
High school graduate	31.5		32.2		23.7		23.3
Some college or more	41.8		42.0		67.5		68.1
**Era (survey years)**		0.671				0.237	
2001-2005	23.2		23.6		29.7		30.7
2006-2009	19.1		19.6		18.7		18.7
2010-2013	29.0		28.2		23.7		23.4
2014-2018	28.7		28.7		27.9		27.3

Note: Data from 2001-2018 National Health Interview Survey (NHIS). The sample was restricted to adults with known vital status as of December 31, 2019 and eligible for survival analysis.

© 2022 Yabroff KR et al. *JAMA Health Forum.*

* Sample characteristics used for propensity score matching included age group at the time of survey, survey weight, number of health conditions, sex, marital status, educational attainment, and survey year era. NHIS sample weight was included in the propensity matching algorithm but not presented here.

†Not married includes widowed, divorced, separated, or never married.

|| Conditions included arthritis, asthma, cancer, diabetes, emphysema, heart disease (angina, coronary heart disease, heart attack, other heart condition/disease), high cholesterol, hypertension, and stroke.

© 2022 Yabroff KR et al. *JAMA Health Forum.*
